# Transitional care experiences of caregivers of preterm infants hospitalized in a neonatal intensive care unit: A qualitative descriptive study

**DOI:** 10.1002/nop2.899

**Published:** 2021-05-05

**Authors:** Ruo Han Ma, Qing Zhang, Zhi Hong Ni, Hai Tao Lv

**Affiliations:** ^1^ Neonatal Intensive Care Unit Children's Hospital of Soochow University Suzhou China; ^2^ Medical College of Soochow University Suzhou China; ^3^ Nursing Department Children's Hospital of Soochow University Suzhou China

## Abstract

**Aim:**

To describe the transitional care experiences and nursing needs of caregivers of preterm infants hospitalized in neonatal intensive care units (NICUs).

**Design:**

A qualitative descriptive study.

**Methods:**

We conducted semi‐structured interviews with the 24 caregivers of preterm infants admitted to Children's Hospital, Soochow University. All data were collected by a trained and experienced interviewer. The caregivers' experiences were described using qualitative content analysis.

**Results:**

Six Five themes emerged from the analysis: (a) uncertainty about the disease; (b) anxiety due to restricted visitation; (c) exhaustion from overwork; (d) emotional depression; (e) low care ability; (f) a variety of channels for help and a positive response. This study provides a basis for understanding the needs of their caregivers so that effective coping strategies can be implemented. Nurses' education and practice should focus on understanding the real experiences of the parents of preterm infants during transitional nursing.

## 1 INTRODUCTION

A preterm infant is a baby born before 37 weeks of gestation. According to data from the World Health Organization, each year, more than 15 million infants are born preterm worldwide, and about 10% of these or about 1,170,000 preterm infants are born in China (Zou et al., 2014). Because of their physical immaturity, preterm infants have a high incidence of complications such as hypoglycaemia, apnoea, infection, feeding difficulties, necrotizing enterocolitis and anaemia (Caldas et al., 2018). Preterm infants are usually admitted to the neonatal intensive care unit (NICU) after birth. To prevent cross infection, the NICU of children's hospital restricts parents' visits to their children. There are webcams in the ward, and caregivers can use the hospital visiting machine to see their children. Preterm infants can only be discharged when their condition meets the following discharge criteria: stable vital signs; corrected gestational age of 34 weeks; weight greater than 2,000 g along with continuous growth; complete oral feeding; stable temperature and vital signs at room temperature; and if a disease is present, full recovery from the disease or partial recovery until treatment at home is acceptable (Picone et al., 2011). Compared with their peers, preterm children require more intensive care after discharge, which can be challenging and causes considerable stress for the parents and other caregivers (Msall, 2019). The parents of preterm infants often experience more severe negative emotional reactions and psychological pressures than those experienced by the parents of healthy full‐term infants, and thus, they require ongoing care and support (Amorim et al., 2018; O'Donovan & Nixon, 2019; Spittle et al., 2018). Furthermore, the mental state of the parents not only affects their own health but also determines the quality of care they provide to their children. The parents and other caregivers of preterm infants often lack experience in providing such care and lack relevant medical knowledge, which leads to difficulties in caring for such infants (Nilsson et al., 2015). Additionally, improper care may lead to adverse consequences.

Transitional care incorporates a broad range of services designed to ensure the continuity of health care as patients' transition from one healthcare setting to another (Coffey et al., 2017). In the case of preterm infants, transitional care typically aims to ensure that patients continue to receive adequate care as they are transferred from the hospital environment to their family homes, so as to minimize adverse consequences (Liu et al., 2018). An important process of transitional care is skill transfer from healthcare professionals to the parents and other caregivers, specifically the transfer of self‐management skills and necessary nursing techniques. Studies have shown that adequate discharge preparation and transitional care can help the families of preterm infants successfully transition from the hospital to the family and reduce the rate of re‐admission (Boykova & Kenner, 2012). For this purpose, healthcare professionals must provide parents with useful information on disease management, foster ties with the hospital staff, encourage the sharing of experiences and feelings, and conduct home visits. Most caregivers of preterm infants report wanting more support from healthcare professionals (Agarwal et al., 2018). However, few studies have described the transitional care experiences and needs of caregivers of preterm infants hospitalized in NICUs.

When preterm infants are discharged from the hospital, they often have complicated medical problems, such as pulmonary dysfunction, visual and auditory impairment and neurodevelopmental disabilities, including cerebral palsy, global developmental delay, intellectual disability and language impairments (Natarajan & Shankaran, 2016). Preterm infants often require home oxygen use, continued developmental assessments, rehabilitation exercises etc. The admission of preterm infants to the NICU itself is a serious negative event for the parents and causes much psychological trauma to the parents and even affects family relationships.

Garfield et al. (2016) reported that the parents of preterm infants have differing concerns as they transition from the NICU to home. Many of these concerns can be addressed with improved discharge information exchanges and anticipatory guidance. Supporting parents during this stressful and often difficult transition may lead to decreased family stress, improved care and better infant outcomes. After discharge, 16% of families experienced significant challenges with two or more barriers; communication difficulties were the most commonly reported barriers during NICU stays (Enlow et al., 2014). Peacock (2014) reported that deficits in communication and information transfer at the time of discharge remain problematic for the caregivers of preterm infants. A successful hospital discharge must include an accurate instructions for medications and establishment of timely follow‐up appointments with the primary care providers. Therefore, it is necessary to develop a systematic, comprehensive and personalized transitional care programme to help families of preterm infants successfully complete the transition from hospital to family care.

The purpose of this study is to understand the real experiences of the parents of preterm infants during transitional nursing, in order to provide a theoretical basis for constructing a transitional nursing mode for preterm infants.

## 2 METHODS

### 2.1 Ethics statement

The study protocol was approved by the ethics committee of Children's Hospital, Soochow University, Suzhou City, Jiangsu Province, China (approval no., #2016010). The objectives, methods and importance of the study were explained to the caregivers, who were also told that if they so desired, they could leave the study at any point, even after having signed the informed consent form. Confidentiality was strictly maintained during the study. We deleted any identifying information from the interview transcripts and used number codes to refer to the patients.

### 2.2 Study design

This study had a qualitative descriptive design and used semi‐structured, open‐ended interviews to analyse the transitional care experiences and nursing needs of the caregivers of preterm infants. All interviews were conducted between January and June 2018 at Children's Hospital, Soochow University. We used purposive sampling to enrol the caregivers and also included a convenience sample of preterm infants who were treated in the NICU of our hospital between January and June 2018. Our NICU has 100 beds and 2 units for critically ill newborns. It is the highest medical‐level NICU in Suzhou, China. The patients were treated by a multidisciplinary team that included paediatricians, nurses, nurse specialists, psychologists, social workers and dietitians. The inclusion criteria for the preterm infants were as follows: (a) admission to the NICU, (b) 26 weeks < gestational age < 37 weeks and (c) at‐birth Apgar score ≥6 points. Children with congenital malformation, hereditary disease or other serious complications were excluded from the study. Caregivers were eligible for inclusion in the study if they were the child's parents, they were able to clearly express themselves, volunteered to participate in this study and provided written informed consent. Caregivers with a history of schizophrenia were excluded from the study.

### 2.3 Data collection

The interviews were performed in a quiet consulting room of the hospital. Qualitative data‐collection methods included face‐to‐face individual interviews.

A senior researcher (NZH) performed the interviews and trained less experienced co‐workers. NZH is an experienced nurse whose highest credential is a PhD. All the researchers in this study were female and experienced in performing qualitative research.

To create the semi‐structured interview, we consulted five paediatric newborn nurses and referred to systematic reviews of the related literature (Barlow et al., 2018; Gad et al., 2017; Mughal et al., 2017). Initially, a preliminary interview of five caregivers was conducted. The data from the preliminary interviews were not included in this study, but were used to modify the interview structure according to the preliminary outcomes. The final interview that was used in this study included the following items: (a) What did the caregivers already know about preterm infants? (b) Did the nurses tell the caregivers everything they needed to know, and did they want to know anything else? (c) How did the caregivers feel during the transitional care process? (d) What was the most challenging problem encountered during the transitional care process? (e) How did they solve the difficulties encountered? (f) What was the most significant help they needed? To capture the parents' real experiences of transitional care at different time points, the responses of the interviewees were obtained using three separate interviews, conducted 1 week before discharge, 1 week after discharge and 1 month after discharge. We conducted one‐on‐one interviews with the primary caregivers of the preterm infants at all three time points. Only the participant and the interviewer were present during the interviews; no other family members were allowed in the interview room. Each interview lasted 30–40 min. We continuously collected data until no new events occurred, thereby achieving data saturation (FitzGerald et al., 2008). In this study, saturation was reached when no new or contrasting results emerged from the interviews. The researcher used audio recordings to collect the data, and field notes were made after each interview.

For qualitative content analysis (Elo & Kyngas, 2008), the interviews were first transcribed word for word, and then, interview notes were compiled. The second investigator carefully read the transcripts to familiarize themselves with the data and then extracted the most relevant words and phrases to describe the transitional care experiences of the caregivers. The first and third investigators read all the transcripts. The investigators extracted sentences that conveyed the most meaningful information about the experiences and needs of the caregivers. This was followed by preparing the coding sheets, grouping the data, and creating and abstracting categories. Codes were used for the various descriptions, and data categorization was performed multiple times by the investigators, who worked closely together until finally, six main categories were identified. As a confirmatory test, the six categories were shown to the caregivers who all agreed that the results accurately represented their experiences. Data analysis was conducted using the NVIVO software (QST International, Cambridge, MA, USA).

### 2.4 Rigor

We ensured that the results obtained in this study were credible, transferable, consistent and confirmable by using previously described methods, as listed below (Nemetchek et al., 2019; Olsson et al., 2017).

#### 2.4.1 Credibility

We included patients with diverse characteristics in terms of age, sex and level of education.

#### 2.4.2 Transferability

Prior to this study, we strived to establish a trustworthy and friendly relationship with the caregivers. The same interviewers interviewed all caregivers and encouraged them to freely express their feelings, while themselves remaining neutral.

#### 2.4.3 Consistency

All phases of the analysis were described in detail. The duration and number of interviews were increased if the situation demanded it.

#### 2.4.4 Confirmability

Two investigators with limited or no clinical experience with preterm infants analysed the data.

The transcripts were read and re‐read until the researchers became familiar with the overall content. During this time, notes were made about potential codes. Data analysis involved the development of a list of codes that identified any feature of the data that was interesting and noteworthy. An inductive approach was adopted whereby coding was strongly linked to the data. Three researchers independently coded two transcripts, and a good level of inter‐rater agreement was found. Two of the researchers then coded the remaining interviews, and a third researcher was consulted where there were discrepancies. The codes were examined by all three researchers for ways in which they could be grouped to form themes or categories. In the final phase of the analysis, all researchers reviewed the data and agreed upon extracts that were representative examples of the themes that they had identified.

## 3 RESULTS

### 3.1 General information

After the application of the selection criteria, a total of 24 caregivers (18 mothers and 6 fathers) aged between 22–38 years were enrolled in this study. In addition, 14 preterm male infants and 10 preterm female infants, with gestational ages ranging from 28–36 weeks, were included in the study. The general characteristics of the preterm infants and their caregivers are listed in Tables 1 and 2, respectively.

**TABLE 1:** Demographic data of the preterm infants

Variable	*N*	*F* (%)
Gender		
Male	14	58.3
Female	10	41.7
Gestational age (weeks)		
28–32	8	33.3
33–36	16	66.7
Birth weight (grams)		
<1,500	5	20.8
1,501–2,000	13	54.2
>2,000	6	25
Diagnosis		
Jaundice	4	16.7
Asphyxia	5	20.8
Haemolytic disease	3	12.5
NRDS	7	29.2
HIE	5	20.8
Only child		
Yes	16	66.7
No	8	33.3

Abbreviations: HIE, hypoxic ischaemic encephalopathy; NRDS, neonatal respiratory disease syndrome.

**TABLE 2:** Demographic data of the caregivers

Variable	*n*	*F* (%)
Education		
Middle school	4	16.7
Junior college	9	37.5
University	11	45.8
Income (yuan)		
<4,000	5	20.8
4,000–6,000	9	37.5
>6,000	10	41.7
Occupation		
Unemployed	3	12.5
Company worker	8	33.3
Agricultural worker (poor)	4	16.7
Office clerk	9	37.5
Residence		
City	11	45.8
County	13	54.2
Caregiver		
Mother	18	75.0
Father	6	25.0

### 3.2 Theme identification

The data analysis identified the following six themes: (a) uncertainty about the disease, (b) anxiety due to restricted visitation, (c) exhaustion from overwork, (d) emotional depression, (e) low care ability and (f) a variety of channels for help and a positive response (Table 3). Each theme is described below with supporting quotes from the participants.

**TABLE 3:** Superordinate and sub‐themes identified in the analysis

Themes	Sub‐themes
(1) Uncertainty about the developmental outcomes of their infants	Parents showed uncertainty, anxiety, nervousness, fear and other negative emotions during their child's hospitalization
	Parents were concerned about the developmental outcomes of their infants
(2) Anxiety due to leave their newborn children	Parents were not satisfied with the NICU visitation system
	Parents were anxious to leave their newborn children
(3) Exhaustion from overwork	Caregivers showed weariness
	Some preterm babies have physical weakness, which makes it more difficult to take care of them
(4) Emotional depression	The parents of preterm infants who were admitted to NICU will experience acute stress disorder, which is characterized by self‐blame and guilt or lack of personal efficacy
	The repeated admission to the hospital put great pressure on the parents
	Many caregivers were in great pain and felt anxious when their children were born
(5) Low care ability	Many caregivers were first‐time parents. They had no experience with parenting a preterm infant
	Caregivers of preterm infants typically have little knowledge of preterm care
(6) A variety of channels for help and a positive response	Many parents will choose to seek help from books, networks and other channels
	Parents will choose to respond positively to help from elders, friends, books, networks, medical personnel and other channels

### 3.3 One week before discharge

#### 3.3.1 *Theme* *1: Uncertainty about the developmental outcomes of their infants*

Preterm infants have a high incidence of complications, which tends to prolong hospital stays and puts a great pressure on the parents of the children. Some parents showed uncertainty, anxiety, nervousness, fear and other negative emotions during their child's hospitalization. Caregivers showed a sense of uncertainty about the patient's condition.

During the period of hospitalization, I received many phone calls about disease communication from doctors, and I was particularly afraid of the phone call. I was afraid that the doctor would tell me that the child's condition has changed, that he has difficulty breathing, and that he would need a respirator to breathe. (Mother 13)

The doctor said that my child had an ear … hearing problem, and I am afraid she can't hear later, she can't speak later, and fear that she has intellectual impairment in the future. (Father 3)

Parents were concerned about the developmental outcomes of their infants, based on their neonatal stay.

My child is so young, the doctor told me that the child's organ is not fully developed, and there may be dysplasia later. Our family is worried. (Mother 18)

Why are other people's children normal, and my baby unnormal? I wonder if she will lag behind children of the same age and have mental retardation. (Mother 15)

#### 3.3.2 *Theme* *2: Anxiety due to leave their newborn children*

Due to the critical condition of the infants, the NICU restricts parents' visits to their children. Although the parents followed the NICU visitation system, they were not satisfied with these limitations. They hoped for an NICU visitation system that was more humane. A family‐centred model can let caregivers participate in the care of preterm infants and promote their mental health (Kim et al., 2020). Caregivers showed anxiety due to restricted visitation.

I don't know about children's situation. It all depends on doctors and nurses to tell us about the change of children's condition. We see videos of children on visiting machine in family waiting area. I'm very worried about my baby. (Father 4)

I know that children are seriously ill and need isolation treatment. I can't affect the treatment of doctors. I can't visit children. I feel anxious every day and in no mind to do anything. (Mother 7)

The parents were anxious to leave their newborn children, especially the mothers.

The child was hospitalized. I've been in a daze all day. Although I can watch the children's videos on the hospital visiting machine, I really want to hold her in person. (Mother 9)

During pregnancy, I have established a deep feeling with my child. Now I can't see him. I really miss my child. (Mother 12)

### 3.4 One week after discharge

#### 3.4.1 *Theme* *3: Exhaustion from overwork*

After preterm infants are discharged, caregivers need to face the daily care of the children, which entails a multitude of manual tasks, such as breastfeeding, changing diapers, feeding medicines and bathing. Many parents feel powerless and fatigued during this period. In this study, caregivers showed weariness.

It's hard to be a mother. I have to make milk powder and feeds for my baby every night. I have to do rehabilitation exercises for her every day. Her dad works; he's too busy and can't help me. I really feel too tired. (Mother 8)

Every day is busy … no time to take care of myself. My baby often cries in the evening. Sometimes, I only sleep 2–3 hours a night. I just can't stand it anymore, but I cannot fall; the baby is so small. What can he do if I fall down? (Mother 6)

Some premature babies have physical weakness and dyspepsia after birth, which makes it more difficult to take care of them.

My child suffers from indigestion after birth. He is so small, I do not dare to touch him. I fear he will choke when feeding him. He often vomits after eating milk. I'm afraid his malnutrition will lead to poor growth. (Mother 16)

I like a machine in order to take care of the baby from morning till night, breastfeeding, and medicine feeding. It was difficult for him to finish eating 30 ml of milk. After a while, he vomited it all out. The bed sheets and clothes were all wet. I had to change it. Otherwise, he'll get sick again after catching a cold. I think I'm going to collapse. (Mother 4)

#### 3.4.2 *Theme* *4: Emotional depression*

The parents of preterm infants who were admitted to NICU will experience acute stress disorder. When there is disagreement among family members, parents deliberately suppress their emotions, and the stress disorder will be more obvious, which is characterized by self‐blame and guilt or lack of personal efficacy.

My wife cried every day when our baby was in the NICU. She worries about her children all day long. I went to work during the day, and I had to take care of them at night, so it's not easy to deal with all kinds of problems. (Father 5)

The baby is my second child. I did not pay attention to the body during pregnancy; otherwise, the baby should not be preterm. Now I'm taking care of the children by myself. I am afraid of affecting the rest of … the other son and the family members. (Mother 10)

Many children were hospitalized again after being discharged from the hospital, and the repeated admission to the hospital put great pressure on the parents.

The child's illness is very serious … in the continuous oxygen inhalation. I wonder if her illness will get worse. My sleep at night has been poor. There is a sense of despair … cannot see the end. (Mother 14)

I am really afraid that the child is getting worse. What should I do if the child is not good? How do our family take it? (Father 2)

Many caregivers were in great pain and felt anxious when their children were born. As time went on, they tended to gradually accept their reality.

At first, I couldn't accept the reality. The people in our family were all healthy. What's the matter? Slowly, I admit this fact, and now, I have worked hard to learn a lot of knowledge about caring for preterm infants, which is very helpful for me to take care of the children. (Mother 11)

#### 3.4.3 *Theme* *5: Low care ability*

The growth and development of premature infants lag behind those of healthy full‐term infants. Preterm infants also have low immunity. Therefore, preterm infants have special care needs. In this study, 17 caregivers were first‐time parents. They had no experience with parenting a preterm infant. They did not have the skills to change their children's diapers and clothes, or bathe their children. In the beginning, they could not take care of their children.

When the child was discharged, I began to try to hold him. He's too small for me to hold him. I didn't know how to hold him. I didn't dare to give him a bath. He was soft and small. What if he slips into the water? (Mother 17)

My wife and I are parents for the first time. We don't have experience with children. We don't know anything about parenting of a preterm infant. Everyone is in a hurry when the baby come home. I really hope that some professionals will give some advice. (Father 6)

Due to their low birth weight, preterm infants need great care. The methods to care for preterm infants are quite different from those used for healthy full‐term infants. Moreover, small omissions in care can lead to adverse consequences; for example, improper feeding may lead to vomiting and suffocation in preterm infants, yet caregivers of preterm infants typically have little knowledge of preterm care.

My child often spits. He suffocates as soon as he eats milk. I don't know what to do. My friends are inexperienced too. (Father 1)

I'm looking for information on the parenting of preterm infants on the Internet now, but some of the information on the Internet is unprofessional, and I don't know how to do it. (Mother 3)

### 3.5 One month after discharge

#### 3.5.1 *Theme* *6: A variety of channels for help and a positive response*

Facing various challenges in child care and disease care, many parents will choose to seek help from books, networks and other channels.

My wife likes to see some related books, what should be done, like a baby's red buttocks, what should be noticed when she is sucking … all she looks at in the book. (Father 1)

I often communicate with others on WeChat. Many friends who are parents will answer some of my questions. Sometimes people talk about how to take care of their children. Although they are not as professional as healthcare professionals, they can also solve difficulties. (Mother 1)

I will search the Internet for some parenting knowledge. After all, it is impossible to call the healthcare professionals frequently. They are very busy. (Mother 5)

Caregivers considered that they had insufficient knowledge and skills to care for their babies and expressed a desire to learn from professionals. Faced with all kinds of perplexities in child care and disease care, most parents will choose to respond positively to help from elders, friends, books, networks, medical personnel and other channels.

As the child was in hospital, I consulted the doctors and nurses for some knowledge of caring for very‐low‐weight babies, and the nurses were patient to guide me. I hope that they will continue to help and care for us. I'm going to take care of my children carefully, so that he can grow up healthily. (Mother 2)

Nurses are the people I trust, and they are very enthusiastic when I go back to the hospital to consult the nurses about the baby's problems. (Mother 14)

## 4 DISCUSSION

Nursing is a key issue for the care of preterm infants admitted to the NICU and for their subsequent rehabilitation. The parents of these children are typically under great pressure, especially during hospitalization (Lubbe, 2018). For new parents, the hospitalization delays their entry into a parental role. Parents experience many types of discomforts, which continue even after the children have returned to their families. During this transition period, parents need to adapt to their roles as soon as possible and take on the task of caring for their children. If parents cannot adapt to their roles, then this will result in further stress and negative emotions. These emotions not only affected their physical and mental health but also had a negative impact on family harmony. Consistent with the results of other researchers (Garfield et al., 2016; Granero‐Molina et al., 2019), we found that the parents of preterm infants reported negative emotions and sleep disorders. During hospitalization, the main causes of stress were the NICU environment and the separation from the newborn children. When the children gradually improved and returned to their families, the sources of parental stress changed; distress was now mainly caused by the burden of family care and difficulties in parenting.

This study identified differences in the stresses experienced by the parents of preterm infants. In addition, the mothers of premature babies experienced varying degrees of self‐blame and guilt. They believed that they were somehow responsible for their children's ill health. Furthermore, failure to carry out childcare tasks made mothers feel frustrated. This finding is in line with the findings reported by Lundqvist et al. (2019). Their studies have shown that more than 90% of mothers of children discharged from the hospital feel uneasy in their parenting life (Lundqvist et al., 2019).

The traditional postpartum practice of Zuo Yue Zi presents Chinese mothers with additional difficulty in establishing early physical interactions with their infants. Meanwhile, the findings from this study revealed that practices and policies still existed to separate parents from infants and limit their involvement in infant care, despite the advocacy of family‐centred care practices existing in NICUs in China (Yu et al., 2020).

In this study, the fathers of the children also experienced increased mental pressure. This is consistent with findings from Chen et al. (2019). In addition to economic pressures and emotional depression, they reported needing to take care of the children and provide support for the mothers. An increasing number of studies are focussing on the stress experienced by the fathers of preterm infants. Some studies have shown that although fathers are less likely to develop depression than mothers, they are at still at risk for depression (Chen et al., 2019). Fathers faced more decision‐making pressures than mothers during hospitalization. The mothers reported feeling some weakness after the childbirth and were more likely to need to recuperate at home. During this time, the fathers were usually responsible for making treatment‐related decisions and providing informed consent for various treatment plans. Thus, the fathers experienced specific problems during the hospitalization of their preterm infants owing to their dual role in caring for both the preterm infants and the mothers. They worried about the safety of both the sick children and their wives and were unable to properly control some of their negative emotions, owing to which they were prone to anxiety (Prouhet et al., 2018).

Family members can influence each other in terms of emotional, behavioural and cognitive aspects. Furthermore, the findings from the present study indicated parents showed negative emotions such as anxiety and depression when the child was ill. This is consistent with findings from Mughal et al. (2017). At the same time, parents, as the main support of their children, directly affect the quality of life of the children. Good coping ability of the parents and family harmony are conducive to the recovery of the children. Nurses play an important role in supporting the parents of premature infants. Nurse participation not only provides support to the parents but also helps to successfully complete the transition from the NICU to the family. This study showed that parents felt that healthcare professionals did not value the parents' role in this transition.

Therefore, considering the psychological pressures on parents during different periods of care, nurses should comprehensively assess the situation of the children, the parents' needs, and the family environment and function and take different measures to help parents cope with various problems and alleviate the pressure on the parents.

Emotional support is the basis for building correct beliefs and an important factor for effective nurse–patient communication (Tandberg et al., 2018). Psychological care for parents should include emotional support, allowing parents to express their feelings. If we listen to parents, we can obtain a general understanding of the parents' personality characteristics, inner feelings, cultural background, social relations and complex experiences (Silveira et al., 2018).

Almost all of the parents in this study could not tolerate the long separation from their children and hoped that the visiting times would be extended or the visitation system would be changed. It was another finding from this study. Although remote devices such as visiting machines have been installed in the NICU, the parents were not satisfied with seeing only video images of their children without sound. They hoped to see the treatment of their children in a more intimate manner and communicate with doctors and nurses face to face (Villamizar‐Carvajal et al., 2018).

Usually in the NICU, parents cannot accompany preterm infants all the time, which can result in parents feeling isolated in their parental roles (Heo & Oh, 2019). This study showed that the emotional needs of mothers during their children's hospitalization exceeded their information needs, and they were more willing to participate in the daily care of the children. Nurses were regarded as a bond between mother and child and could promote the development of early mother–child relationships (Spittle et al., 2016). In order to avoid iatrogenic interference in the mother–child relationship, we should try to make up for the missing role of the mother in the hospital environment. For example, nurses can implement kangaroo mother care if the condition of the child permits this to create opportunities for mother–child contact during a stable transition period. As a unique form of communication, mother‐to‐child communication has far‐reaching impacts on the physical, psychological and intellectual development of the child. However, the implementation of the mother‐to‐child interaction programme is often hindered by preterm infants' protective isolation, treatment needs, NICU pattern settings and other factors. With the optimization of NICU pattern and the development of high‐quality nursing, these difficulties will be gradually overcome, and mother‐to‐child communication will gradually take place.

Through information dissemination and behaviour intervention, health education helps individuals and groups to master relevant knowledge, establish health concepts, and voluntarily adopt educational activities and processes conducive to healthy behaviour and lifestyle. Systematic health education not only improves the mental and emotional confusion caused by a lack of knowledge but also makes treatment and nursing more satisfactory and prevents complications. Early education is an effective way to alleviate parental stress. The use of early family education programmes for parents can enhance their confidence and promote family relationships (Khurana et al., 2016). Early education for the parents of preterm infants focusses on understanding the children's needs. For example, by guiding parents to intervene with their children, parents can understand the behaviours of preterm infants, special reflexes, and visual and auditory characteristics. Moreover, they learn how to be competent in the role of a parent, confirm their children's unique behaviours and even provide other parents with information about caring for their children. Postdischarge education should focus on childcare and rehabilitation. In addition, the use of a clinical health pathway in early education can make the health education work of nurses targeted, planned and predictable. With the development of information technology, an increasing number of parents of preterm infants think it is necessary to share their experiences through social networks and other channels. They look for people with similar experiences to seek social support, and many do so within a week of admission. Fathers are mainly concerned about the medical risks of their children, while mothers are more likely to share their emotional reactions. The 24‐hr availability of the Internet facilitates social network support. In addition to providing health education support, parents can also relieve stress by sharing their stories and reading others' stories (Lv et al., 2019).

Healthcare professionals should be the organizers and coordinators of effective peer support projects. Parents of preterm infants with successful experiences and follow‐up needs should be carefully selected. Evidence‐based interventions should be adopted to establish standardized peer support measures, such as training time, training methods, training forms and the proportion of peers to participants, which are dominated by professionals. In addition, promoting communication and friendship between parents after discharge is also an effective method of postdischarge support. NICU medical staff should make use of existing medical resources to organize peer support in cooperation with community health services.

Transitional nursing requires not only the participation of nurses with solid professional knowledge, skilled clinical skills and strong adaptability, but also the support and cooperation of healthcare professionals from multiple disciplines. Healthcare professionals begin this work after preterm infants are discharged from the hospital and work with their caregivers to determine nursing objectives. Each nursing plan is specially designed according to the personal conditions of the preterm infants. Healthcare professionals' guidance to caregivers during the transitional period is mainly embodied in the following principles: (a) Enhancing caregivers' understanding of disease information. Nurses focus on helping caregivers understand disease problems, so that the caregivers can identify the disease when it occurs. During each follow‐up visit, healthcare professionals need to review the implementation of the discharge guidance plan to ensure that caregivers truly understand the health and disease information conveyed to them and effectively implement the nursing plan. (b) Assisting caregivers in managing health problems and preventing disease progression. The caregivers of preterm infants are encouraged to implement care tasks at home, especially, their own health management.

## 5 IMPLICATIONS FOR PRACTICE

Data obtained from this study can serve as a starting point towards developing transitional care for preterm infants. Major problems to providing transitional care of preterm infants in this context have been described. In response to this, strategies can be developed.

Education and training on transitional care for preterm infants are essential to the development of this service. Additionally, developing regulatory frameworks that govern practice and assess clinical competence of transitional care providers is essential to facilitate safe practice and quality care. Findings from this study can also help policymakers develop clinical practice guidelines to inform transitional care for preterm infants.

Healthcare professionals should be more aware of parents' various needs in transitional care and of their important role as constant caregivers. Neonatal care should be specifically designed to supply positive support and necessary strategies for parents to strengthen their confidence in parenting infants.

## 6 LIMITATIONS

A potential limitation of the study is the small number of interviews performed. We interviewed the caregivers of preterm infants 1 week before discharge, 1 week after discharge and 1 month after discharge. In future studies, we will conduct a long‐term and sustained study of the caregivers of preterm infants. However, the interviews were rich in content and included a diverse study population in terms of cultural and social backgrounds. The information gathered started to repeat after 24 interviews, signalling saturation regarding the major themes. Nevertheless, we acknowledge that minor aspects might not have been sufficiently covered or that other participants from different contexts may have differing views. The strengths of this study are the inclusion of a transitional care concept, a solid theoretical background, a thoroughly validated interview guide and a rigorous analysis supported by an interdisciplinary research team. Transition of care represents an important phase in chronic diseases management. An effective transition maintains continuity of care and presents better clinical outcomes (Bert et al., 2020). Therefore, we believe that our findings provide a theoretical basis for constructing a transitional nursing mode for preterm infants.

## 7 CONCLUSIONS

We have conducted a qualitative descriptive study involving semi‐structured interviews to learn the transitional care experiences of 24 caregivers of preterm infants. While the children were admitted to the NICU, parents reported that the separation from their children was difficult to bear and that they felt like they were under great pressure. Therefore, healthcare professionals should strive to understand the needs of caregivers and provide them with relevant medical information to boost their confidence. In addition, care plans should be developed with both preterm infants and their caregivers in mind to enhance nursing and emotional support and minimize stress. The study provides a theoretical basis for constructing a transitional nursing mode for preterm infants.

## CONFLICT OF INTEREST

The authors declare no conflict of interest.

## AUTHOR CONTRIBUTIONS

All authors participated in the study design. MRH: Clinical data collection. All investigators: Data analysis. NZH and LHT: Writing and revision of the draft and subsequent manuscripts. ZQ: Drafting and revision of the manuscript. All authors read and approved the final manuscript.

## Data Availability

The data that support the findings of this study are available from the corresponding author upon reasonable request.
